# The umbilical cord mesenchymal stem cell‐derived exosomal lncRNA H19 improves osteochondral activity through miR‐29b‐3p/FoxO3 axis

**DOI:** 10.1002/ctm2.255

**Published:** 2021-01-13

**Authors:** Litao Yan, Gejun Liu, Xing Wu

**Affiliations:** ^1^ Department of Orthopedics, Shanghai Tenth People's Hospital, School of Medicine Tongji University Shanghai PR China

**Keywords:** chondrocytes, exosomes, long noncoding RNA, umbilical cord mesenchymal stem cells

## Abstract

**Background:**

Our previous study revealed that the exosomal lncRNA H19 derived from umbilical cord mesenchymal stem cells (UMSCs) plays a pivotal role in osteochondral regeneration. In this study, we investigated whether the exosomal lncRNA H19 could act as a competing endogenous RNA (ceRNA) to potentiate osteochondral activity in chondrocytes.

**Methods:**

Dual‐luciferase reporter assay, RNA pull‐down, RNA immunoprecipitation (RIP), and fluorescence in situ hybridization (FISH) were carried to verify the interaction between miR‐29b‐3p and both lncRNA H19 and the target mRNA FoxO3. Chondrocytes were treated with UMSC‐derived exosomes, which highly expressing lncRNA H19 expression, followed by apoptosis, migration, senescence, and matrix secretion assessments. An in vivo SD rat cartilage defect model was carried out to explore the role and mechanism of lncRNA H19/miR‐29b‐3p.

**Results:**

UMSCs were successfully identified, and exosomes were successfully extracted. Exosomes exhibited the ability to transfer lncRNA H19 to chondrocytes. Mechanistically, exosomal lncRNA H19 potentiated osteochondral activity by acting as a competing endogenous sponge of miR‐29b‐3p, and miR‐29b‐3p directly targeted FoxO3. Intra‐articular injection of exosomes overexpressing lncRNA H19 could promote sustained cartilage repair; however, this effect could be undermined by miR‐29b‐3p agomir.

**Conclusions:**

Our study revealed a significant role in the development of strategies against cartilage defects for UMSC‐derived exosomes that overexpress lncRNA H19. Exosomal H19 was found to promote chondrocyte migration, matrix secretion, apoptosis suppression, as well as senescence suppression, both in vitro and in vivo. The specific mechanism lies in the fact that exosomal H19 acts as a ceRNA against miR‐29b‐3p to upregulate FoxO3 in chondrocytes.

## Graphical abstract

Exosomes derived from UMSCs exhibited the ability to transfer lncRNA H19 to chondrocytes.

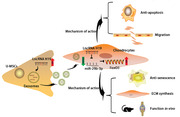

**Abbreviations**
ceRNA: competing endogenous RNA
FISH: fluorescence in situ hybridization
HE: hematoxylin and eosin
OA: osteoarthritis
RIP: RNA immunoprecipitation
Saf‐O: Safranin‐O/fast green
SA‐β‐Gal: senescence‐associated β‐galactosidase
TB: toluidine blue
UMSCs: umbilical cord mesenchymal stem cells

## 1 INTRODUCTION

Osteochondral defects of the knee after a traumatic event remain a significant treatment challenge. Because of the avascular structure and limited self‐renewal capacity of the hyaline articular cartilage, chondral injuries without suitable treatment may result in degeneration and ultimately progress to end‐stage osteoarthritis (OA).^1^ Healing of osteochondral defects remains a clinical challenge with significant research endeavors directed at resurfacing techniques for the management of focal osteochondral defects, including autologous chondrocyte implantation,^2^ microfracture treatments,^3^ scaffold implantation procedures,^4^ and cell‐based therapies.^5^

The field of stem cell therapies has ushered in novel methodologies. Its application in animals has been proven to have successful short‐ and long‐term results.^6^ However, the further development of stem cell strategies for humans is hampered by the lack of a complete understanding of the underlying mechanisms by which osteochondral defects heal. The paracrine effect is acknowledged to be the therapeutic function of stem cells, and exosomes are the main functional molecules involved.^7^ Exosomes are nanosized microvesicles (50‐150 nm in diameter) that contain various noncoding RNAs (miRNAs, lncRNAs, and circRNAs), and are reported to be viable options for the treatment of injured tissues. In addition, exosomes have the advantages of lower immunogenicity and tumorigenic potential compared with that of stem cell transplantation.^8^

We have demonstrated that exosomes derived from umbilical cord mesenchymal stem cells (UMSCs) could be used as natural carriers of the lncRNA H19 and that high level of lncRNA H19 in exosomes could enhance proliferation and prevent apoptosis of chondrocytes. However, the specific mechanism of exosomal H19 (H19‐Exos) in chondrocytes remains unclear. Bioinformatic analysis suggests miR‐29b‐3p/FoxO3 as the downstream signaling pathway of lncRNA H19.^9^, ^10^ FoxO3 plays a key role in development, aging, and longevity. This role is believed to be directly related to the control of cellular homeostasis.^11^ Several studies have demonstrated that lncRNA H19 could act as a competing endogenous RNA (ceRNA) to sponge miR‐29b‐3p in various cancers.^12^, ^13^, ^14^ Recently, Tan et al^15^ found that H19‐Exos derived from fibroblast‐like synoviocytes could alleviate the progression of OA. However, to date, there has been no study on lncRNA H19/miR‐29b‐3p/FoxO3 in chondrocytes.

In this study, we assumed that lncRNA H19 delivered from UMSCs to chondrocytes could competitively bind to miR‐29b‐3p to relieve its repression of the target gene FoxO3, thereby enhancing chondrocyte migration and matrix synthesis and suppressing apoptosis and senescence. Our study suggests a novel mechanism underlying H19‐Exos in cartilage repair, indicating that the lncRNA H19/miR‐29b‐3p/FoxO3 axis might be a therapeutic target for posttraumatic focal cartilage defects.

## 2 METHODS

### 2.1 Ethics statement

This study was conducted under the approval of the Animal Ethical Committee of Shanghai Tenth People's. All animal care and surgical techniques were strictly complied with the Declaration of Helsinki.

### 2.2 Cell culture and isolation of exosomes

Chondrocytes (lot.339995, Beina Biology Research Institute, China) were purchased and maintained in DMEM containing 10% FBS (10099‐141, Gibco, MD). UMSCs (UC‐MSC‐7530, Shanghai Wonderful Medical Science Co. Ltd, China) were purchased and maintained in Dulbecco's Modified Eagle Media containing 10% exosome‐free FBS. Chondrocytes and UMSCs were all incubated in an incubator of 5% CO_2_ and 95% air at 37°C.

Exosomes were isolated based on the procedure according to Théry et al.^16^ Culture supernatants were first passed through a 0.22‐μm filter and then centrifuged at 110 000 × *g* for 70 minutes to pellet exosomes. Exosome pellets were added 5 mL PBS to wash and harvested by centrifugation at 110 000 × *g* for 70 minutes again.

### 2.3 Transmission electron microscopy

Exosomes were fixed with 3% (w/v) glutaraldehyde and 1% osmium tetroxide for 2 hours, respectively. The exosomes were dropped on the copper grid and negatively dyed with 2% uranyl acetate for 1 minute. Then, exosomes were air‐dried and observed under transmission electron microscopy.

### 2.4 Nanoparticle tracking analysis

The ZetaView instrument (Particle Metrix GmbH, Germany) was used to measure the size distribution exosomes. Exosomes were diluted in solution buffer and added for nanosight tracking measurement.

### 2.5 Exosome uptake assay

Exosomes were labeled with PKH67 (Green, Sigma‐Aldrich, MO). Briefly, 100 μL exosome suspension was mixed with 10 μL PKH67 (1:25 in Diluent C) for 10 minutes at 37°C. And 1 mL of 0.5% BSA was used for the termination of staining, and the exosomes were re‐extracted by ultracentrifugation (110 000 × *g* for 70 minutes). The chondrocytes were cocultured with PKH67‐labeled exosomes (10 μg/mL) overnight. Antifade Mounting Medium with DAPI (P0131, Beyotime, China) was used to stain nuclei. The images were obtained from the fluorescence microscope (Leica Microsystems, Germany).

### 2.6 Western blotting

Chondrocytes were lysed with immunoprecipitation lysis buffer (Sigma‐Aldrich, MO) to extract total protein. BCA Protein Assay Kit (P0010, Beyotime, China) was used for the quantification of total protein. Protein extracts were subjected to 10% SDS‐PAGE, and transferred to polyvinylidene fluoride membranes. After blockade of 3% BSA, the membranes were incubated with primary antibodies FoxO3 (ab109629, Abcam, UK, 1:1000), COL2A1 (NB‐600‐844, Novus, MO, 1:1000), Sox9 (ab185966, Abcam, UK, 1:5000), ACAN (A11691, ABclonal, China, 1:500), ADAMTS5 (ab41037, Abcam, UK, 1:1000), Runx2 (ab236639, Abcam, UK, 1:1000), Bcl‐2 (ab32124, Abcam, UK, 1:1000), Bax (ab32503, Abcam, UK, 1:1000), PCNA (ab92552, Abcam, UK, 1:1000), and GAPDH (NB100‐56875, Novus, MO, 1:5000) at 4°C for 12 hours, and incubated with HRP goat anti‐rabbit IgG (AS014, ABclonal, China, 1:2000). The membrane was detected using BeyoECL Star (P0018AS, Beyotime, China). GAPDH was used as the internal reference.

### 2.7 Quantitative real‐time PCR (qRT‐PCR)

The total RNA in exosome and chondrocytes was extracted according to instructions of the TRIzol reagent (Invitrogen, CA). The PrimeScript RT Reagent Kit (Takara, China) was used to transcribe RNA into cDNA reversely. The miRNA First‐Strand cDNA Synthesis Kit (Vazyme, China) was used to transcribe miR‐29b‐3p into cDNA.

Amplification reactions were performed by qRT‐PCR system (ABI 7500, Thermo Fisher Scientific, MA) using 1 μL cDNA and 1 μL primer (Sangon Biotech, China). The reaction volume is 20 μL containing amplification primers and SYBR Premix Ex Taq kit (Takara, China). The reactions were carried out under following conditions: denaturation at 95°C for 3 minutes, 40 cycles of denaturation at 95°C for 3 seconds and 60°C for 30 seconds. The exosomal level of lncRNA H19 was normalized to the synthetic miR‐39 (cel‐miR‐39: 5′‐UCACCGGGUGUAAAUCAGCUUG‐3′). The cellular miR‐29b‐3p expression was normalized to U6. The quantification of cellular lncRNA H19 and target genes was normalized to Beta‐2‐microglobulin. All reactions were run in triplicate with each sample. The relative expression was calculated using the 2^−(ΔCtsample − ΔCtcontrol)^ method. The primers are listed in Table S1.

### 2.8 Flow cytometry assay

Annexin V‐FITC/PI Apoptosis Kit (C1062, Beyotime, Shanghai) was used to detect apoptosis. Briefly, 1 × 10^6^ chondrocytes treated with IL‐1β, H19‐Exos or miR‐29b‐3p mimics were collected and washed three times. 195 μL binding buffer, 5 μL Annexin V‐FITC, and 10 μL propidium iodide were added step by step. After incubation in dark for 30 minutes, apoptosis was detected by a BD FACS flow cytometer (BD Biosciences).

### 2.9 Scratch wound assay

Chondrocytes (5 × 10^4^ cells) receiving different treatments (transfection) were seeded in a six‐well plate. The confluent monolayer of chondrocytes was scratched with a P200 pipet tip. Then 1% FBS DMEM‐F12 as well as NC‐Exos or H19‐Exos (10 μg/mL) was added. The migration was marked by the position of cells through successive frames (0, 24, and 48 hours).

### 2.10 Transwell migration assay

Chondrocytes (2 × 10^5^ cells) receiving different treatments were seeded into the transwell insets (8 μm pore size, Corning, NY). Then 500 μL 1% FBS DMEM‐F12 as well as NC‐Exos or H19‐Exos (10 μg/mL) were added to the lower chambers. Chondrocytes were allowed to migrate for 24 hours. Then, the chondrocytes that had penetrated were stained with 0.1% crystal violet. The amount of penetrated cells was counted in three random fields.

### 2.11 Senescence‐associated β‐galactosidase (SA‐β‐Gal) assay

SA‐β‐Gal activity of chondrocytes was measured using Senescence β‐Galactosidase Staining Kit (C0602, Beyotime, Shanghai). Chondrocytes (5 × 10^4^ cells) receiving different treatments were seeded in a six‐well plate overnight and then cocultured with NC‐Exos or H19‐Exos (10 μg/mL) for both 3 and 7 days. Chondrocytes were fixed with 4% paraformaldehyde for 20 minutes and incubated with staining solution at 4°C overnight. The images were captured using a light microscope.

### 2.12 Cell transfection

H19 overexpression plasmid (pcDNA3.1‐H19) and FoxO3 overexpression plasmid (pcDNA3.1‐FoxO3) were purchased from Shanghai Gene Pharma. si‐Rab27a (5′‐CGGAUCAGUUAAGUGAAGAAAdTdT‐3′), miR‐29b‐3p mimic (5′‐UAGCACCAUUUGAAAUCAGUGUU‐3′), and miR‐29b‐3p inhibitor (5′‐AACACUGAUUUCAAAUGGUG‐3′) were purchased from RiboBio (Shanghai, China). UMSCs or chondrocytes (4 × 10^5^) were seeded into six‐well plates. Lipofectamine 3000 transfection reagent (Thermo, MA) was used for cell transfection with pcDNA3.1‐H19 and pcDNA3.1‐FoxO3 plasmids. Lipofectamine RNAiMAX transfection reagent (Thermo, MA) was used for the si‐Rab27a (50 nM), miR‐29b‐3p inhibitor (100 nM), and mimic (20 nM). After incubation for 6 hours, culture medium was refreshed.

### 2.13 Immunofluorescence

Chondrocytes were seeded on glass coverslips overnight, fixed with 4% paraformaldehyde, premeabilized with 0.5% Triton‐X, and then blocked with 1% BSA. The cells were incubated with Col II (1:200, NB600‐844, Novus, MO), Aggrecan (1:100, NB600‐504, Novus, MO), and MMP13 (1:100, NBP1‐45723, Novus, MO) antibodies at 4°C overnight. Chondrocytes were then incubated with goat anti‐rabbit IgG Alexa Fluor 488 (A0423, Beyotime Biotechnology, Shanghai, China) or cy5 dye (P0183, Beyotime Biotechnology, Shanghai, China). Antifade mounting medium with DAPI (P0131, Beyotime, China) was used to stain nuclei.

### 2.14 Fluorescence in situ hybridization (FISH)

LncRNA FISH Probe Mix (RiboBio, Guangdong, China) was used to perform the FISH assay. Briefly, chondrocytes were fixed with 4% paraformaldehyde and treated with protease K, glycine, and acetylation reagents. After prehybridization at 42°C for 1 hour, chondrocytes were hybridized with Cy3‐labeled H19 probes and 488‐labeled miR‐29b‐3p at 42°C for 12 hours. The nucleus was stained with DAPI. 18S and U6 were internal controls in which 18S was distributed in the cytoplasm and U6 in the nucleus.

### 2.15 Dual‐luciferase report assay

The wild and mutant type sequence of H19 and FoxO3 (WT‐MUT‐H19, WT‐FoxO3, MUT‐FoxO3) was designed and synthesized based on the predicted binding sites. These sequences were cloned into psiCHECK2 luciferase reporter vector. Vectors were transfected with 30 nmol miR‐29b‐3p mimic or negative controls (GenePharma, Shanghai, China) in 293T cells. After 48 hours incubation, 293T cells were lysed and incubated with luciferase solution (Promega, CA). Luciferase activity was detected by a dual‐luciferase reporter system (Promega, CA). Finally, the rates of firefly to Renilla were calculated as the relative luciferase activity.

### 2.16 RNA immunoprecipitation (RIP) assay

Magna RIP RNA‐binding protein immunoprecipitation kit (Millipore, Bedford, MA) was used to conduct the RIP assay. The cells were lysed in which 10% cell extract was input, and the other part coprecipitated with anti‐AGO2 (ab186733, Abcam, UK) or IgG (ab200699, Abcam, UK). The magnetic beads‐antibody complex was washed three times in RIP wash buffer. After detaching with proteinase K buffer, the levels of lncRNA H19 and miR‐29b‐3p were measured by qRT‐PCR.

### 2.17 RNA pull‐down

The chondrocytes were lysed (Ambion Company, Austin, TX) and incubated with biotinylated RNA probe for 12 hours at 37°C. Streptavidin‐coated magnetic beads were then added to cell lysate for 4 hours. Nonspecifically bound RNAs were removed by washing buffer, and the bounded RNA was purified by Trizol. The enrichment of lncRNA H19 was determined by qRT‐qPCR. Relative levels of H19 were normalized to the levels of input.

### 2.18 In vivo protocol and histological analysis

Forty 200 g female SD rats were used in this study. A model of unilateral cartilage defect was created on the femoral trochlear grooves used by drill bit (1.5 mm diameter). After the operation, free walk, and access to food and water were allowed without immobilization. One week after the surgery, these rats were randomly divided into four groups: defects treated with PBS, NC‐Exos, H19‐Exos, and H19‐Exos combination of miR‐29b‐3p agomir. The total solution containing 200 μL PBS or NC‐Exos (1 mg/mL) or H19‐Exos (1 mg/mL) were injected every week. The miR‐29b‐3p agomir (5 nmol) were injected every 4 weeks. These rats were euthanized in two batches at fourth and eighth weeks. The samples were harvested for gross morphologic analysis by three blinded observers according to ICRS macroscopic assessment^17^ (Table S2). Histochemical staining included hematoxylin and eosin (HE), toluidine blue (TB), and Safranin‐O/fast green (Saf‐O). Type I and II collagens were used for immunohistochemical staining. The histologic grading scale reported by Wakitani et al^18^ was used to assess the therapeutic effects of cartilage defect repair (Table S3).

### 2.19 Magnetic resonance imaging analysis

After gross examination, the samples were processed for magnetic resonance imaging analysis, as described in our previous study^9^. Briefly, the joints were scanned by a 3.0 T MRI scanner (GE Healthcare, IL). The sequence was as follows: repetition = 1500 milliseconds, echo = 12 milliseconds, scan = 4:25 minutes, field of view = 120 mm, acquisition matrix = 512 × 256 pixels, and slice thickness = 1.5 mm.

### 2.20 Statistical analysis

Comparisons between two groups were performed using an unpaired Student's *t*‐test. Comparisons among multiple groups were conducted by one‐way analysis of variance (ANOVA) with Tukey's post hoc test. Statistical analysis was performed by GraphPad Prism version 8.0. A *P* < .05 was considered statistically significant.

## 3 RESULTS

### 3.1 lncRNA H19 serves as a sponge for miR‐29b‐3p in chondrocytes

**FIGURE 1:**
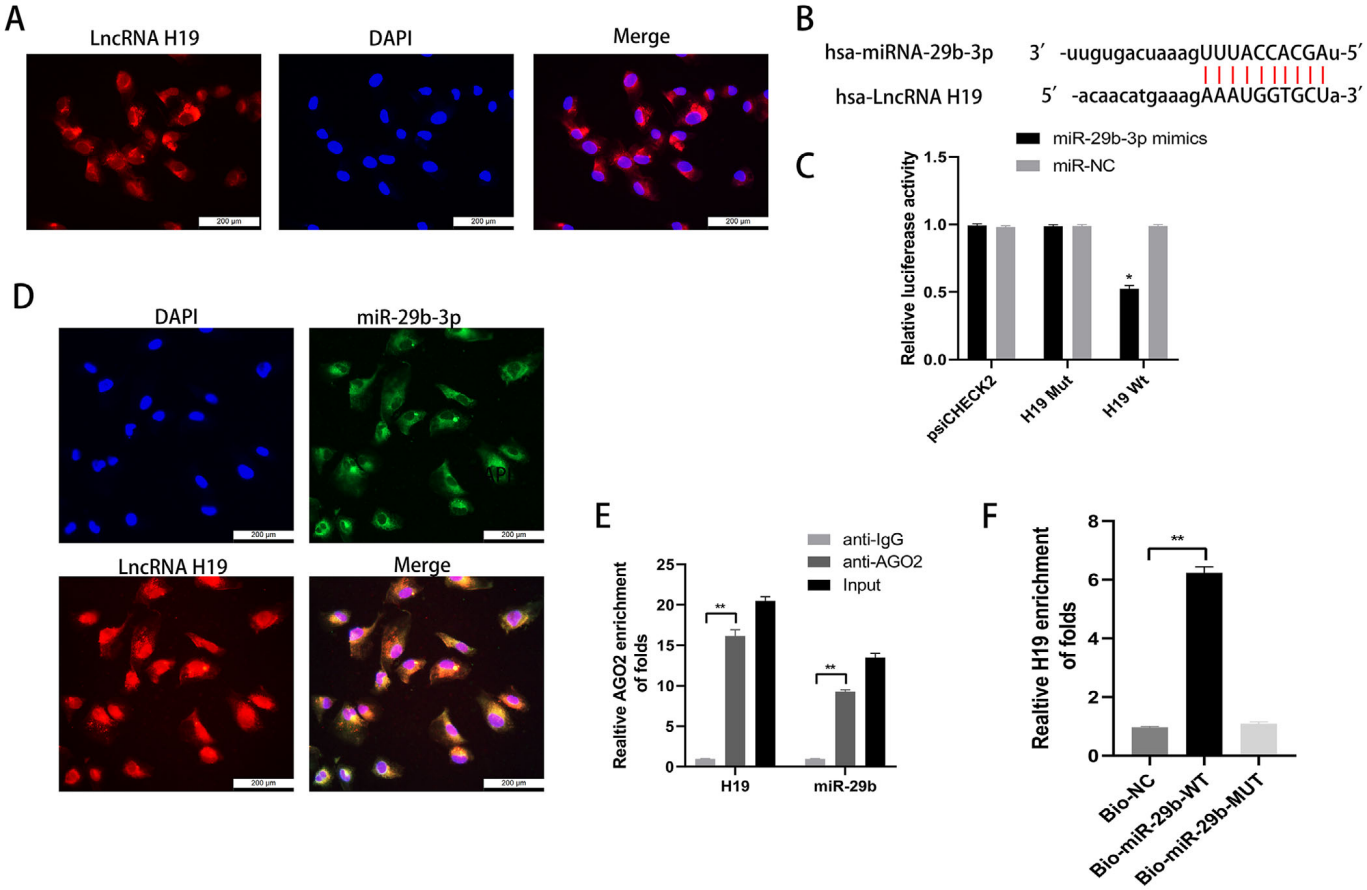
The lncRNA H19 serves as a sponge for miR‐29b‐3p in chondrocytes. A, The subcellular localization of lncRNA H19 was verified by FISH analysis; scale bar: 200 μm. B, The binding sequences of lncRNA H19 and miR‐29b‐3p were predicted by bioinformatics analysis. C, The binding of lncRNA H19 and miR‐29b‐3p was confirmed by the dual‐luciferase reporter assay. *P < .05 compared with the miR‐NC group. D, Colocalization of lncRNA H19 and miR‐29b‐3p by FISH analysis; scale bar: 200 μm. E, The binding efficiency of lncRNA H19 and miR‐29b‐3p to Ago2 protein was detected by RIP. **P < .01 compared with the vector group. F, The relationship between lncRNA H19 and miR‐29b‐3p was tested by RNA pull‐down. *P < .05 compared with the Bio‐NC group. The data are shown as mean ± SD (n = 3)

FISH analysis was performed to detect the molecular mechanisms of lncRNA H19. The result indicated the abundant expression of lncRNA H19 in the cytoplasm of chondrocytes with localization in the nucleus (Figure 1A). We then investigated the ability of H19 to bind to miRNAs. Bioinformatic analysis was performed (miRanda, Targetscan, DIANA), and miR‐29b‐3p was found to harbor a complementary binding sequence for lncRNA H19 (Figure 1B). Moreover, a dual‐luciferase reporter assay was conducted (Figure 1C). The putative or mutated miR‐29b‐3p binding site was cloned into luciferase reporter plasmids. The cotransfection of miR‐29b‐3p mimic and WT‐H19 reporter significantly reduced luciferase activity compared with MUT‐H19. It was found that lncRNA H19 was able to bind to miR‐29b‐3p, and this was further confirmed in the FISH experiment, which showed the colocalization of the two (Figure 1D). Furthermore, RIP and RNA pull‐down assays were performed. As shown in Figure 1E, H19 and miR‐29b‐3p were substantially and simultaneously enriched by anti‐Ago2 compared with IgG (*P* < .01), suggesting that miR‐29b‐3p could be a target of lncRNA H19. Results of RNA pull‐down assay showed that H19 RNA could be pulled down by bio‐miR‐29b‐WT, whereas the corresponding bio‐miR‐29b‐MUT had no effect on H19 expression (*P* < .01, Figure 1F).

### 3.2 UMSCs were successfully identified, and exosomes were successfully extracted

**FIGURE 2:**
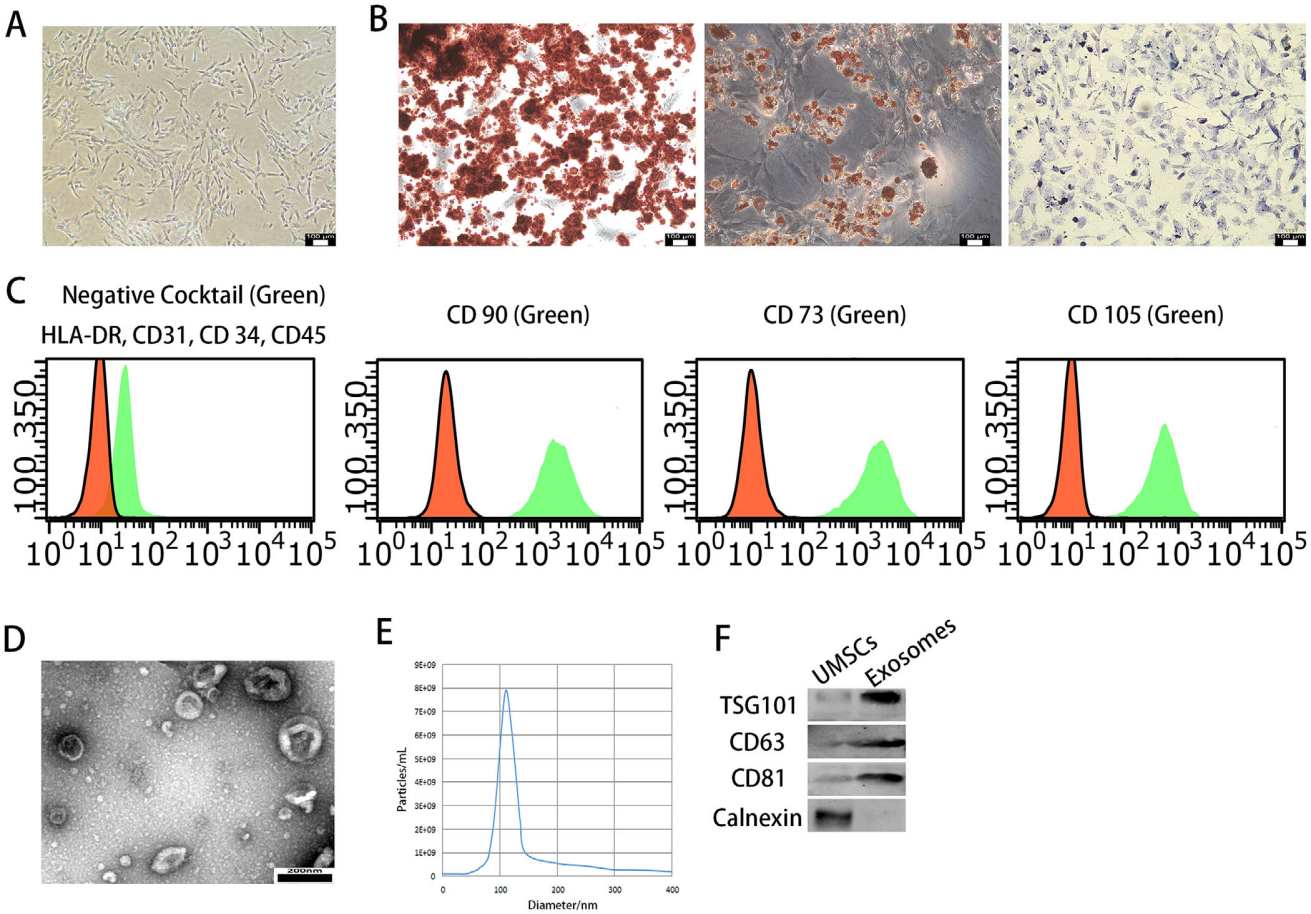
UMSCs were successfully identified and exosomes were successfully extracted. A, Morphological observation of UMSCs (×100). B, UMSCs exhibited multiple osteogenic, adipogenic, and chondrogenic differentiation ability. C, The surface antigen expression of UMSCs was detected by flow cytometric analysis. D, Morphology of exosomes under TEM; scale bar: 200 nm. E, The diameter and concentration of exosomes by nanosight. F, The expressions of TSG101, CD63, CD81, and calnexin in exosomes were detected by Western blot analysis. The data are shown as mean ± SD (n = 3)

UMSCs exhibited a typical spindle shape and exhibited a homogeneous fibroblastic morphology with clear nuclei (Figure 2A). They displayed multiple osteogenic, adipogenic, and chondrogenic differentiation abilities (Figure 2B). Flow cytometry revealed that UMSCs were negative for HLA‐DR, CD31, CD34, CD45 and positive for CD73, CD90, CD105 (Figure 2C). Transmission electron microscopy of exosomes revealed a cup‐shaped morphology (Figure 2D). Nanosight analysis verified that the diameter of exosomes was approximately 120 nm (Figure 2E). The exosomes were positive for CD63, CD81, TSG101 and negative for calnexin (Figure 2F).

### 3.3 Exosomes derived from UMSCs could transfer lncRNA H19 to chondrocytes

**FIGURE 3:**
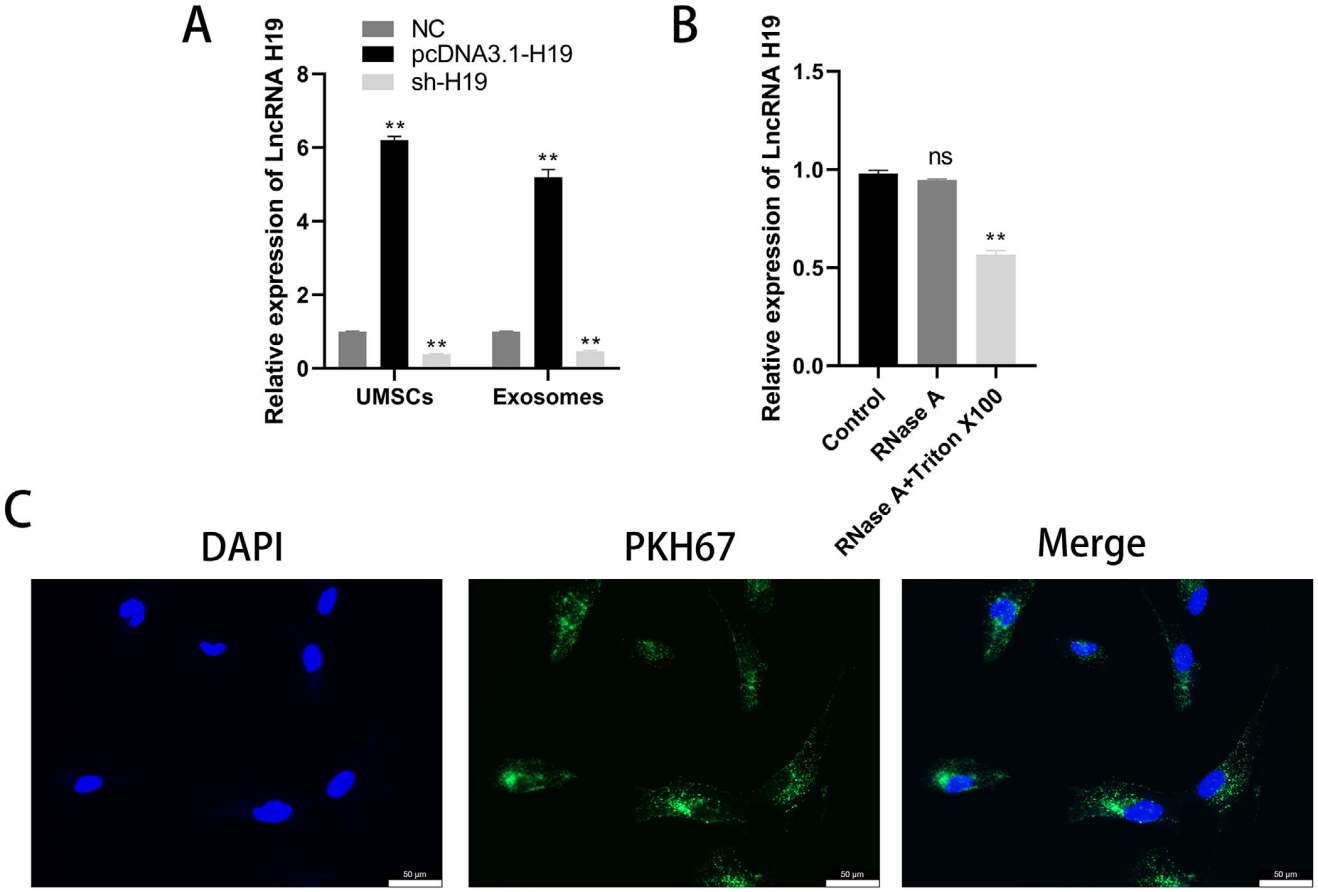
Exosomes derived from UMSCs could transfer lncRNA H19 to chondrocytes. A, The expression of lncRNA H19 in UMSCs and exosomes was detected by qRT‐PCR. **P < .01 compared with the control group. B, The expression of lncRNA H19 in exosomes treated with RNase A or Triton X‐100. **P < .01 compared with the control group. C, Uptake of PKH67‐labeled exosomes in chondrocytes was observed by confocal fluorescence microscopy; scale bar: 50 μm. The data are shown as mean ± SD (n = 3)

To investigate whether the exosomal lncRNA H19 derived from UMSCs could be transferred to chondrocytes in vitro, UMSCs were first transfected with pcDNA3.1‐H19. The expression of lncRNA H19 was higher than the control at both UMSCs (H19‐MSCs) and exosomes derived from UMSCs (H19‐Exos) in Figure 3A (*P* < .01). When exosomes were treated with both RNase A and Triton X‐100, not just RNase A, the level of lncRNA H19 significantly decreased, indicating that lncRNA H19 was protected by membranes of exosomes against RNase degradation (*P* < .01, Figure 3B). Chondrocytes were cocultured with exosomes labeled with PKH67 and as shown in Figure 3C, the chondrocytes tested positive for green fluorescence. This suggested that exosomes released by UMSCs had the ability to transfer lncRNA H19 to chondrocytes.

### 3.4 Exosomal lncRNA H19 derived from UMSCs potentiated osteochondral activity by directly inhibiting miR‐29b‐3p

**FIGURE 4:**
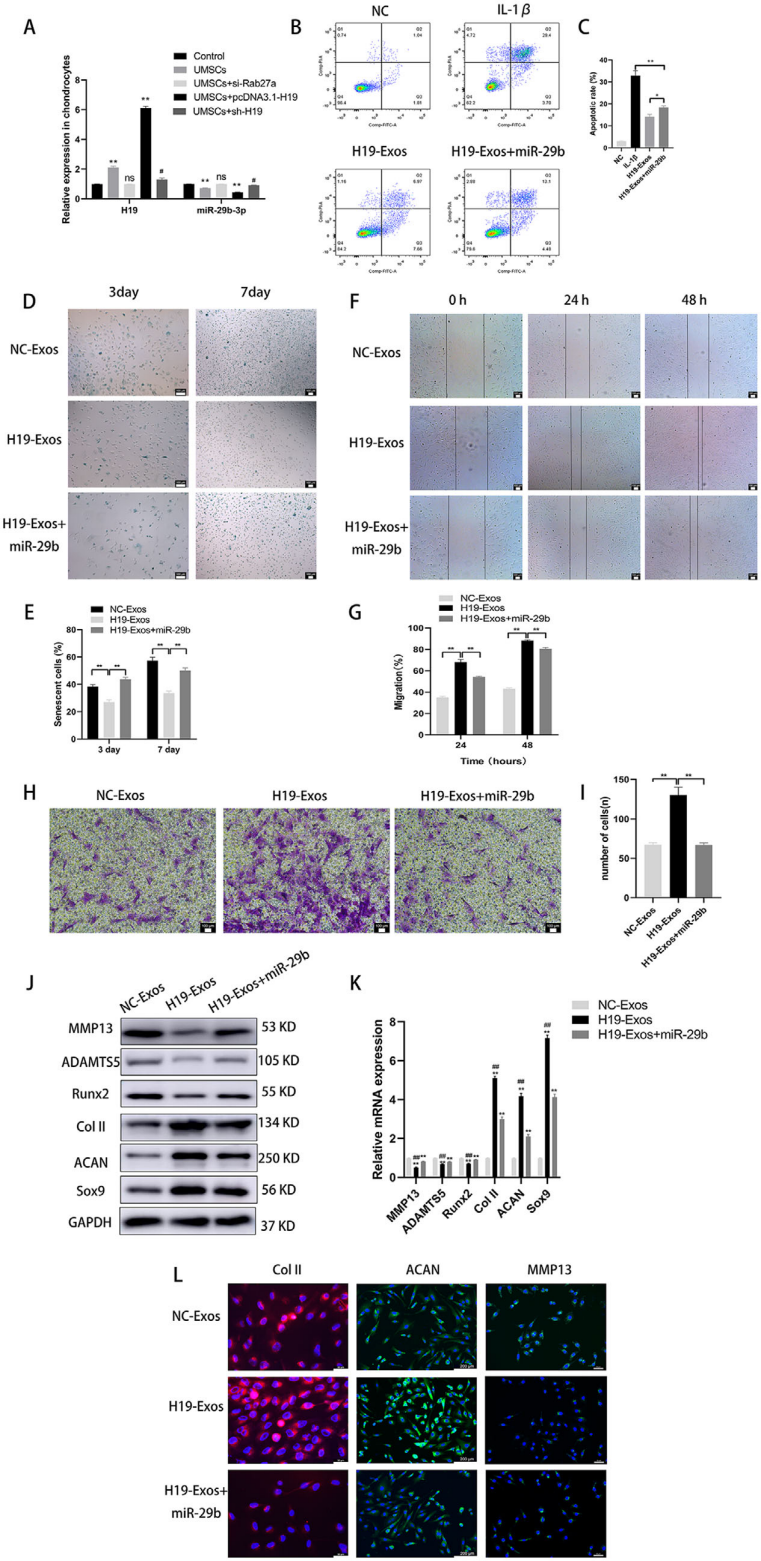
UMSC‐derived exosomal lncRNA H19 potentiates osteochondral activity by directly inhibiting miR‐29b‐3p. A, Expression of lncRNA H19 and miR‐29b‐3p in chondrocytes, which were cocultured with UMSCs or UMSC‐derived exosomes treated with si‐Rab27a, or MSC‐derived exosomes overexpressing H19, or MSCs transfected with sh‐H19. *P < .05 and **P < .01 compared with the control group; ^#^ *P* < .05 compared with the UMSCs. B and C, Chondrocytes were treated with H19‐Exos with or without miR‐29b mimics and stimulated by IL‐1β (10 ng/mL) challenge for 24 hours. Apoptosis was assessed by flow cytometry. *P < .05, **P < .01. D and E, Light microscopy images and quantitative analysis of chondrocytes stained with the senescent marker SA‐β‐Gal in monolayer culture; scale bar: 100 μm. *P < .05, **P < .01. F and G, Light microscopy images and quantitative analysis of scratch wound assays. H and I, Light microscopy images and number of transmitted cells in the transwell migration assay. *P < .05, **P < .01. J and K, The protein and mRNA level of genes associated with chondrocytes (MMP13, ADAMTS5, Runx2, Col II, aggrecan, and Sox9). L, Immunofluorescence for MMP13, Col II, and aggrecan. The data are shown as mean ± SD (n = 3)

To detect whether in vitro transferred H19‐Exos could positively regulate miR‐29b‐3p in chondrocytes, qRT‐PCR was performed to detect the expression of lncRNA H19 and miR‐29b‐3p in chondrocytes cocultured with different kinds of UMSCs. The siRNA‐Rab27a was used to inhibit exosome release from the UMSCs. There was a significant increase in lncRNA H19 in chondrocytes cocultured with H19‐UMSC, whereas the opposite effect occurred with miR‐29b‐3p, which showed a decreased expression (*P* < .01, Figure 4A).

To determine whether lncRNA H19 functions in osteochondral activity via the direct interaction with miR‐29b‐3p, chondrocytes were cocultured with normal exosomes (NC‐Exos), exosomes highly expressing H19 (H19‐Exos), or H19‐Exos with a miR‐29b‐3p mimic. As shown in Figure 4B,C, the coculture of H19‐Exos decreased the rate of chondrocyte apoptosis in the presence of IL‐1β. However, the antiapoptotic activity of H19‐Exos was markedly attenuated by the overexpression of miR‐29b‐3p.

We assessed the SA‐β‐Gal in chondrocytes at 3 and 7 days, and it was found that chondrocytes could spontaneously senesce under normal culture conditions (Figure 4D,E). Treatment with H19‐Exos significantly could decrease the amount of SA‐β‐Gal‐positive chondrocytes (*P* < .01). However, the protective effects of countering the senescence of chondrocytes were abolished after miR‐29b‐3p overexpression (*P* < .01).

A scratch assay indicated that the migration of chondrocytes in the H19‐Exos group appeared much higher than in the other groups (*P* < .01, Figure 4F,G); however, the migration ability was attenuated by transfection with miR‐29b‐3p mimic (*P* < .01). Ultimately, a transwell assay showed that the number of transmembrane chondrocytes in the H19‐Exos + miR‐29b‐3p group was lower than that in the H19‐Exos group (*P* < .01, Figure 4H,I).

In the presence of H19‐Exos, the protein and mRNA levels of cartilage‐enriched markers (Col II, aggrecan, and Sox9) decreased, while levels of hypertrophic and inflammatory markers (MMP13, ADAMTS5, and Runx2) increased in the chondrocytes coinfected with miR‐29b‐3p mimic, as compared with those cocultured with H19‐Exos alone (Figure 4J,K). Immunofluorescence analysis confirmed that the expression of Col II and aggrecan decreased, while the expression of MMP13 increased in chondrocytes coinfected with miR‐29b‐3p mimic (Figure 4L).

These results demonstrate that the beneficial effects of lncRNA H19 on cartilage could be blocked by miR‐29b‐3p and that lncRNA H19 functions by targeting miR‐29b‐3p in vitro.

### 3.5 FoxO3 was a direct target of miR‐29b‐3p

**FIGURE 5:**
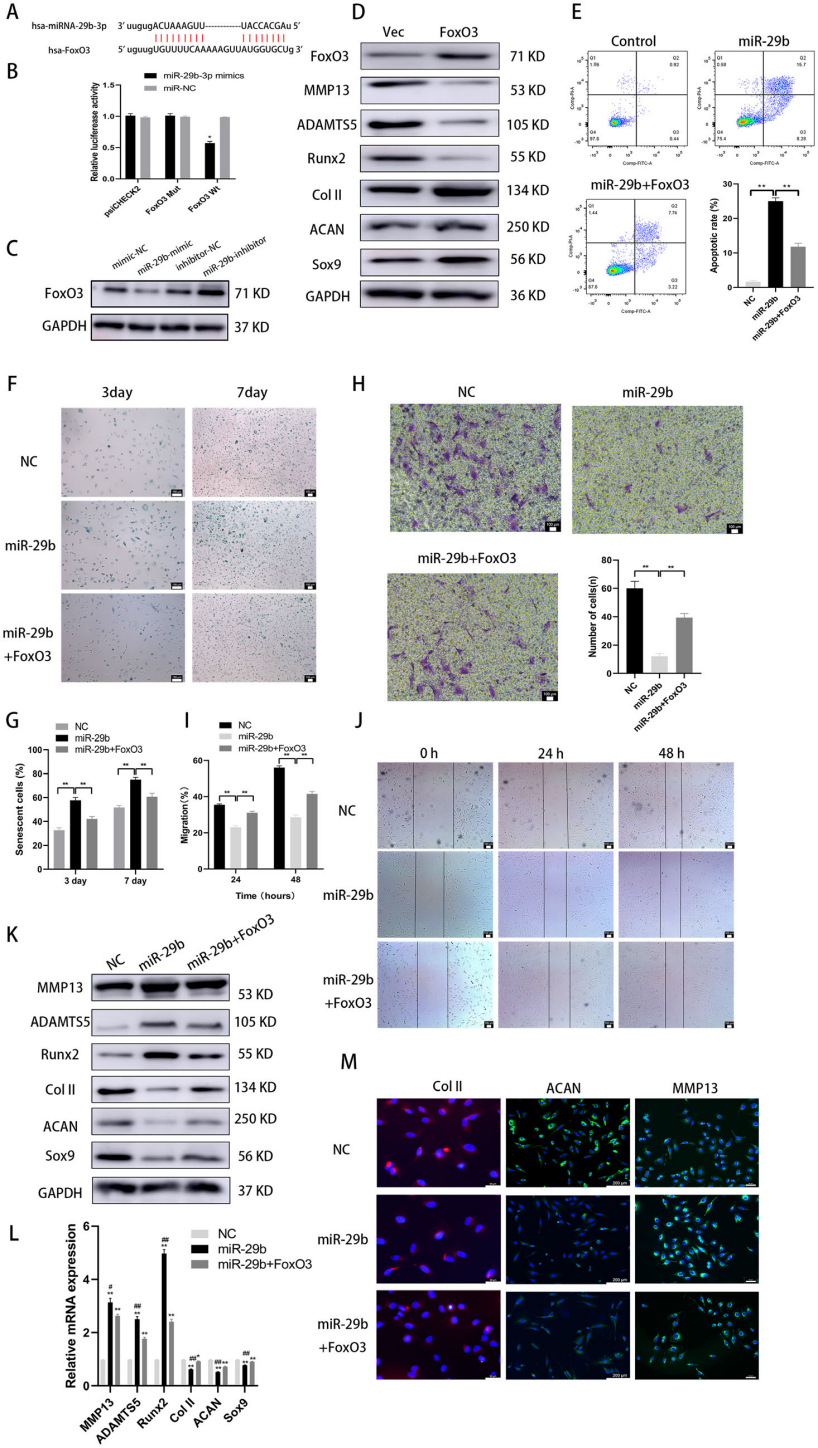
FoxO3 was a direct target of miR‐29b‐3p. A, Binding sequences of miR‐29b‐3p and FoxO3 were predicted by bioinformatics analysis. B, Binding of miR‐29b‐3p and FoxO3 was confirmed by the dual‐luciferase reporter assay. *P < .05 compared with the miR‐NC group. C, miR‐29b‐3p overexpression reduced FoxO3 level, while miR‐29b‐3p inhibition increased FoxO3 level. D, FoxO3 overexpression increased the protein level of Col II, aggrecan, and Sox9 and decreased the level of MMP13, ADAMTS5, and Runx2. E, Chondrocytes were treated with miR‐29b mimics with or without overexpressing FoxO3 and stimulated by IL‐1β (10 ng/mL) challenge for 24 hours. Apoptosis was assessed by flow cytometry. F and G, Light microscopy images and quantitative analysis of chondrocytes stained with the senescent marker SA‐β‐Gal in monolayer culture; scale bar: 100 μm. H, Light microscopy images and number of transmitted cells in the transwell migration assay. I and J, Light microscopy images and quantitative analysis of scratch wound assays. K and L, Protein and mRNA level of genes associated with chondrocytes (MMP13, ADAMTS5, Runx2, Col II, aggrecan, and Sox9). M, Immunofluorescence for MMP13, Col II, and aggrecan. The data are shown as mean ± SD (n = 3; *P < .05, **P < .01)

LncRNAs can act as ceRNAs to regulate expression of target mRNAs by sponging miRNAs. Bioinformatic prediction was performed (miRanda, Targetscan, DIANA), and the results confirmed that FoxO3 was a putative target of miR‐29b‐3p (Figure 5A).

A luciferase assay demonstrated that the miR‐29b‐3p mimic markedly inhibited the luciferase activity of WT‐FoxO3 compared to the negative control group, while MUT‐FoxO3 abolished the repressive effect of the miR‐29b‐3p mimic (Figure 5B). In addition, the miR‐29b‐3p mimic diminished the expression of FoxO3, while the miR‐29b‐3p inhibitor exhibited opposite effects (Figure 5C). These results verified the strong negative correlation with FoxO3.

Moreover, FoxO3 overexpression increased the protein levels of Col II, aggrecan, and Sox9 and decreased the levels of MMP13, ADAMTS5, and Runx2 (Figure 5D). These results demonstrated that the miR‐29b‐3p targeted FoxO3 and that endogenous FoxO3 expression was closely related to the osteochondral activity of chondrocytes.

We designed a rescue assay to confirm whether the miR‐29b‐3p/FoxO3 pathway modulated osteochondral activity in chondrocytes. The infection with the miR‐29b‐3p mimic significantly promoted apoptosis (Figure 5E), accelerated aging (Figure 5F,G), and reduced the migration ability of chondrocytes. Moreover, the protein and mRNA levels of MMP13, ADAMTS5, and Runx2 were increased, and the levels of Col II, aggrecan, and Sox9 were decreased in the chondrocytes (Figure 5H‐J). These effects were undermined by the overexpression of FoxO3 (Figure 5K,L). Immunofluorescence analysis confirmed that the expression of Col II and aggrecan increased, while MMP13 expression decreased in the chondrocytes coinfected with FoxO3 (Figure 5M).

Collectively, these results demonstrated that downregulation of miR‐29b‐3p could enhance osteochondral activity of chondrocytes by negatively regulating FoxO3.

### 3.6 Role of lncRNA H19/miR‐29b‐3p in SD rat cartilage defect model

In previous studies, we have demonstrated that H19‐Exos plays an important role in cartilage repair in SD rats. In this study, we aimed to determine whether miR‐29b‐3p undermines the effects of H19‐Exos, to prove that H19‐Exos acts as a sponge to adsorb miR‐29b‐3p in vivo. PBS, NC‐Exos, H19‐Exos, and H19‐Exos with miR‐29b‐3p agomir were intra‐articularly injected into the cartilage defect model.

**FIGURE 6:**
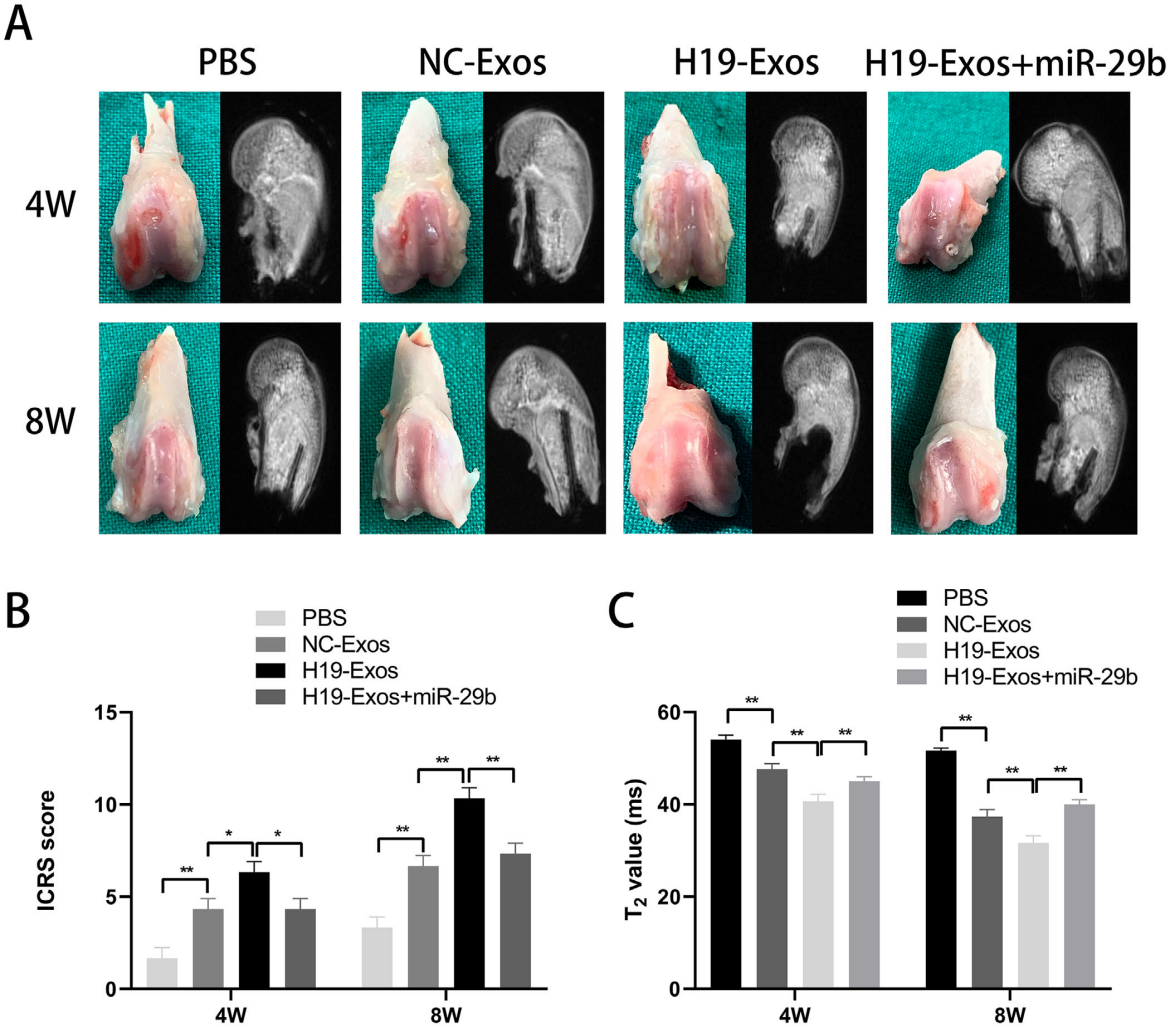
Macroscopic and MRI images of the regenerated tissues at 4 and 8 weeks. A, Representative macroscopic and MRI images of the regenerated tissues at 4 and 8 weeks. B, ICRS macroscopic scores. C, T2 mapping scores. The data are shown as mean ± SD (n = 5; *P < .05, **P < .01)

Macroscopic and magnetic resonance images of samples at 4 and 8 weeks are shown in Figure 6A. The ICRS scores were used to quantify the macroscopic images and indicated that the reparative effects of H19‐Exos were significantly greater than those of the NC‐Exos and H19‐Exos + miR‐29b‐3p groups at both 4 and 8 weeks (Figure 6B). The T2 mapping values were used to quantify the magnetic resonance images, and the results showed that the scores of H19‐Exos were lower than those of both NC‐Exos and H19‐Exos + miR‐29b‐3p groups at 4 and 8 weeks (Figure 6C).

**FIGURE 7:**
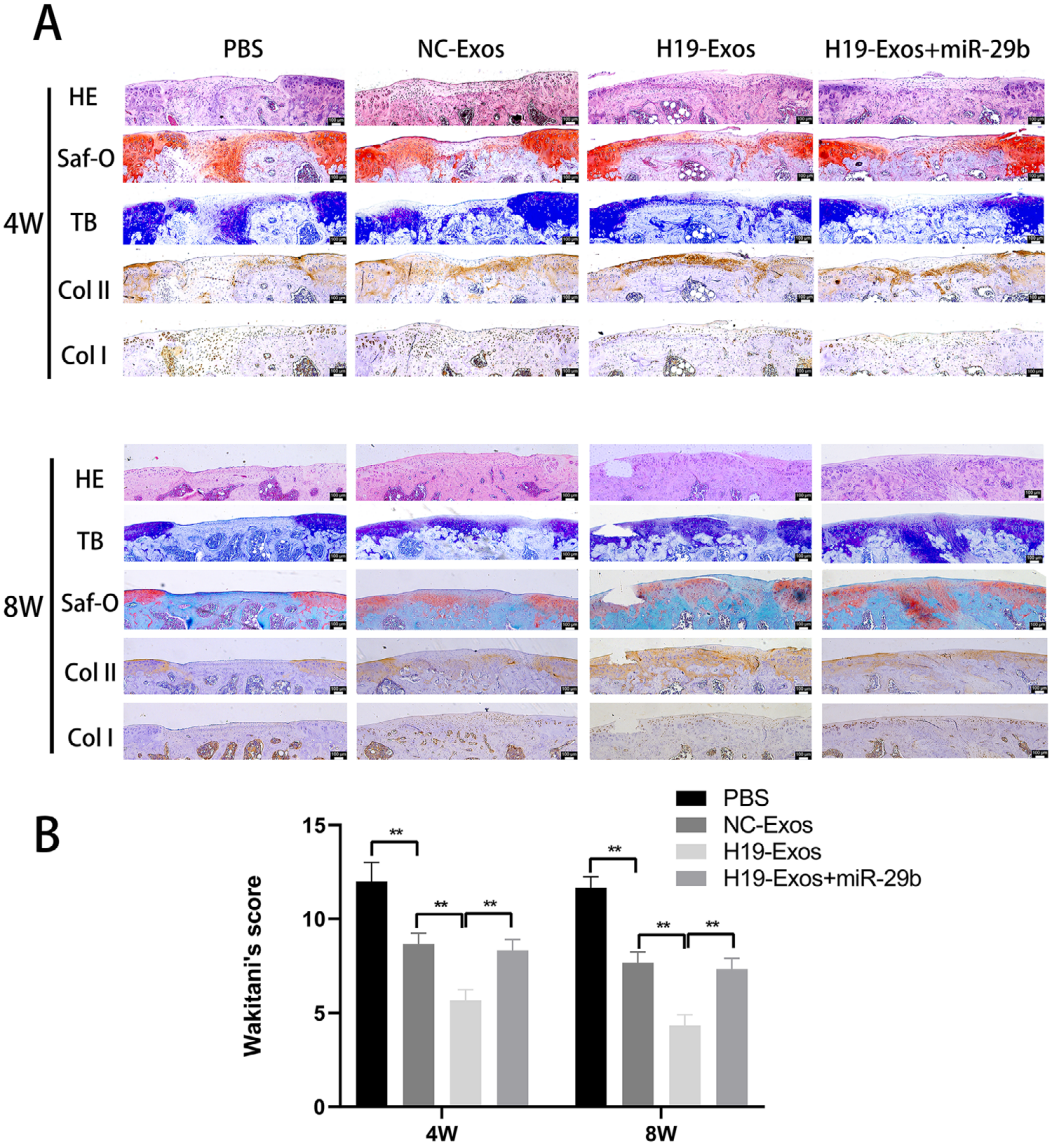
Histologic evaluation of cartilage repair at 4 and 8 weeks. A, Staining results of hematoxylin and eosin (HE), toluidine blue (TB), Safranin‐O/fast green (Saf‐O), and immunohistochemical staining for type II and I collagens. B, Wakitani scores for the histological sections. The data are shown as mean ± SD (n = 5; **P < .01)

Histological assessment of HE, TB, and Saf‐O revealed that the H19‐Exos group showed the most well‐organized tissues and glycosaminoglycan deposition (Figure 7A). Immunohistochemical staining of collagens I and II revealed that the H19‐Exos group showed the best cellularity, and the neonatal cells appeared chondrocytic (Figure 7A). In addition, we found that miR‐29b‐3p agomir undermined the effects of H19‐Exos on the repair of cartilage defects in vivo (Figure 7A). The Wakitani scores were used to evaluate the quality of repaired tissues, and it was found that the scores of H19‐Exos were lower than those of both NC‐Exos and H19‐Exos + miR‐29b‐3p groups at 4 and 8 weeks (Figure 7B). Taken together, these results demonstrate the positive effects of H19‐Exos on cartilage repair through miR‐29b‐3p.

## 4 DISCUSSION

At present, no effective treatments have been approved for the clinical treatment of posttraumatic focal cartilage defects.^19^ To develop effective therapeutics, it is important to investigate the underlying mechanisms of cartilage repair. LncRNAs are considered endogenous, tissue‐specific, and conserved molecules in regulating diverse biological processes by adsorbing miRNAs.^20^

lncRNA H19 is a highly conserved sequence that has been well studied in the field of cancer.^21^ Additionally, it has been associated with cartilage and skeletal muscles, as well as the processes of embryonic growth and stem cell differentiation. lncRNA H19 is abundantly expressed in embryonic tissues, indicating its potential function in determining the future of embryonic cells. Pang et al^22^ found that lncRNA H19 participated in the regulatory function of cartilage differentiation in adipose tissue‐derived stromal cells by regulating STAT2. Downregulation of lncRNA H19 is also closely related to developmental dysplasia and the progression of dislocation of the hip (DDH). Ning et al^23^ revealed that lncRNA H19 binds to let‐7 and promotes the proliferation of chondrocytes. Wang et al^24^ simulated the DDH environment in vitro and found that lncRNA H19 inhibited cartilage degeneration through the miR‐283‐5p/Dusp5 axis.

Further, lncRNA H19 may exert dual and opposing effects on articular chondrocytes. MiR‐675 is encoded by the lncRNA H19, and both of its arms have opposing effects on MMP1 and MMP13, the enzymes related to the breakdown of CLO2A1 in chondrocytes.^25^ Recently, Zhang et al^26^ reported that lncRNA H19 was upregulated in OA chondrocytes. It is possible that the H19/miR‐106a‐5p/TCF4 axis may be responsible for the progression of OA. Yang et al^27^ confirmed that silencing lncRNA H19 prevented chondrocyte apoptosis, promoted proliferation, and downregulated MMP13 levels.

The conclusions of the aforementioned studies differ from ours in terms of the effect of lncRNA H19 on cartilage, and we believe there are three main reasons for these differences. First, it is controversial whether lncRNA H19 is upregulated in OA cartilage. In Zhang et al's^28^ high‐throughput microarray of five OA cartilage tissues, we found no difference in the expression of lncRNA H19 through bioinformatic analysis. Wang et al^29^ analyzed 230 Chinese patients with OA and found that the risk of OA was associated with the specific single‐nucleotide polymorphisms of the H19 rs217727 loci. This may influence the expression of lncRNA H19 in chondrocytes. Through heterogeneity tests, Wang et al^30^ proved that the risk of H19 rs2067051 on OA was more relevant in younger females than males (BMI ≥ 25, age < 60). Therefore, more OA samples are needed to analyze the expression of lncRNA H19 that may be related to genetic variation, gender, race, and age. Second, our study focused on H19 in exosomes derived from UMSCs, rather than lncRNA H19 at the base level of chondrocytes. The study by Tan et al^15^ determined that H19 in fibroblast‐like synoviocyte‐derived exosomes could alleviate OA and, therefore, is in agreement with this study. H19‐Exos promoted cell viability and migration, and protected against extracellular matrix degradation by regulating the miR‐106b‐5p/TIMP2 axis. Third, our research purpose was to analyze the effect of H19‐Exos on the osteochondral activity of normal chondrocytes, which rarely involves OA cartilage. Moreover, our in vivo animal model is a cartilage defect model representing posttraumatic focal cartilage defects.

A variety of miRNAs have been previously reported to be involved in cartilage damage and repair. Chen et al^31^ demonstrated that the overexpression of miR‐29b‐3p in chondrocytes significantly inhibited proliferation, induced apoptosis, and suppressed cell cycle entrance from the G0/G1 to the S phase. Furthermore, he verified the loss of cartilage caused by miR‐29b‐3p antagomir in vivo. MiR‐29b‐3p was found to be highly expressed in synovial fluid and peripheral blood mononuclear cells among OA patients.^32^ Sox9, PGRN, and COL2A1 have been shown to be negative regulators of miR‐29b‐3p.^31^, ^33^, ^34^ Recent studies revealed that lncRNA H19 could act as a sponge to inhibit miRNA‐29b‐3p in bladder cancer cells,^12^ colorectal cancer cells,^13^ and lung adenocarcinoma cells.^35^

**FIGURE 8:**
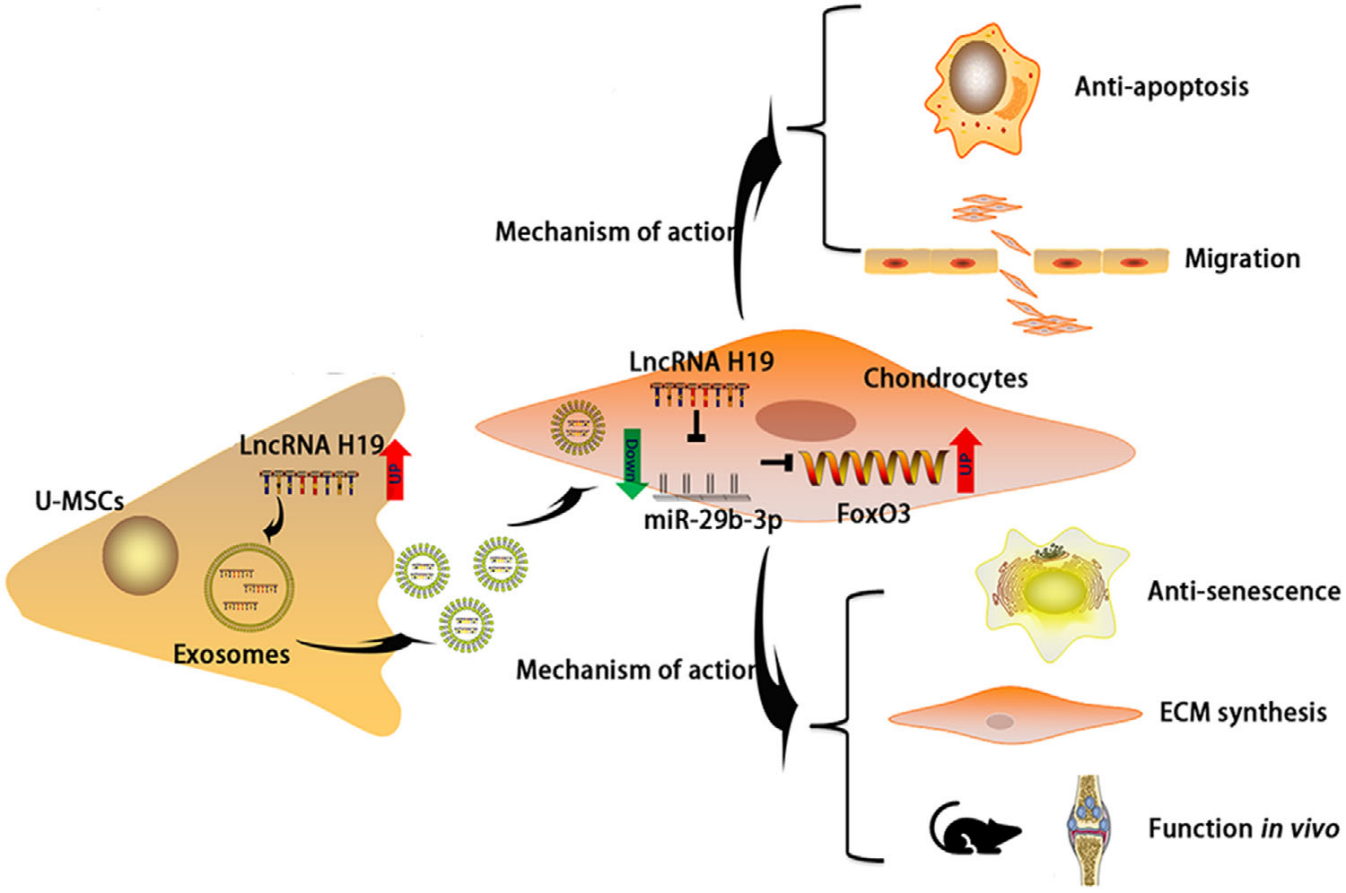
Schematic presentation of working hypothesis

In our study, lncRNA H19 contained a conserved miR‐29b‐3p target site, and this was validated via dual‐luciferase, FISH, RIP, and RNA pull‐down assays. We used gain‐of‐function and loss‐of‐function approaches to demonstrate that the expression of FoxO3 was negatively regulated by miR‐29b‐3p, a mechanism reported herein for the first time (Figure 8).

It has been indicated that the expression of FoxO was decreased in OA human cartilage.^36^ The FoxO family has four members (FoxO1, 3, 4, and 6), of which FoxO1 and FoxO3 are highly expressed in cartilage.^37^ During the differentiation process of MSCs toward chondrogenesis, FoxO3 expression is strongly upregulated from day 21 onwards and in mature chondrocytes.^38^ In addition, overexpression of FoxO3 could increase levels of Phlpp1, an essential enzyme for proper chondrocyte function.^39^ Chondrocyte viability was significantly reduced by transfection with si‐FoxO3 in an in vitro experiment, potentially due to the reduced levels of antioxidant and autophagy proteins.^40^ Knockdown of FoxO3 increased apoptosis and reactive oxygen species levels in chondrocytes.^41^ After surgical joint injury or aging of cartilage, the total and phosphorylated levels of FoxO3 were reported to be decreased.^42^ Matsuzaki et al^43^ found that Col2Cre‐FoxO3 KO mice resulted in severe OA after 18 months of age. Taken together, these studies demonstrated crucial roles for FoxO3 in postnatal cartilage development, maturation, and maintenance of articular cartilage homeostasis.

## 5 CONCLUSION

Based on the aforementioned findings, this study revealed that exosomes from UMSCs carrying lncRNA H19 could be effectively internalized by chondrocytes. We have identified that H19‐Exos could promote chondrocyte migration, matrix secretion, and suppression of apoptosis and senescence in vitro and in vivo. The specific mechanism lies in the fact that H19‐Exos acts as a ceRNA against miR‐29b‐3p to upregulate FoxO3 in chondrocytes. Exosomes containing elevated levels of lncRNA H19 may serve as the basis for an effective therapeutic strategy for posttraumatic focal cartilage defects.

## AUTHOR CONTRIBUTIONS

Xing Wu designed and provided financial support for the study. Litao Yan performed experiments and wrote the paper. Gejun Liu performed data analysis and provided experimental materials and platforms.

## CONFLICT OF INTEREST

The authors declare that there is no conflict of interest.

## Supporting information

Supporting Information

## Data Availability

The datasets in this study are available from the corresponding author on request.
