# Supplementary material for: The umbilical cord mesenchymal stem cell‐derived exosomal lncRNA H19 improves osteochondral activity through miR‐29b‐3p/FoxO3 axis
Source: Clin Transl Med. 2021 Jan 13;11(1):e255. doi: 10.1002/ctm2.255 (PMC7805401; doi:10.1002/ctm2.255)
Supplement: Supplementary file 1 — Supporting Information [file CTM2-11-e255-s001.docx]

**Table 1**

**Primer sequences for quantitative real-time polymerase chain reaction**

| Gene | Primer | Sequence（5′–3′） |
| --- | --- | --- |
| LncRNA H19 | Forward | GCAAGAAGCGGGTCTGTTT |
|  | Reverse | GCTGGGTAGCACCATTTCTT |
| Sox9 | Forward | GCCTCTACTCCACCTTCACC |
|  | Reverse | GTAGACGGGTTGTTCCCAGT |
| COL II | Forward | ATTGCCTATCTGGACGAAGC |
|  | Reverse | GCAGTGTACGTGAACCTGCT |
| MMP-13 | Forward | GCATTGGCTGAGTGAAAGAGAC |
|  | Reverse | ATGATGAACGATGGACAGATGA |
| ADAMTS5 | Forward | ATGATTCGCCTCGGGGCTC |
|  | Reverse | GCACTCTCCGAAGGGGATCT |
| Aggrecan | Forward | ACCAGACTGTCAGATACCCC |
|  | Reverse | CATAAAAGACCTCACCCTCC |
| Runx2 | Forward | ATGATTCGCCTCGGGGCTC |
|  | Reverse | GCACTCTCCGAAGGGGATCT |
| FoxO3 | Forward | ATCTGAGCAGGTTGCTCCAC |
|  | Reverse | GGCCCTTTACACTGTGAGCC |
| miR-29b-3p | Forward | UAGCACCAUUUGAAAUC |
|  | Reverse | GTGCAGGGTCCGAGGT |
| Beta-2-microglobulin | Forward | ATCTGAGCAGGTTGCTCCAC |
|  | Reverse | GGCCCTTTACACTGTGAGCC |

**Table 2**

**International Cartilage Repair Society (ICRS) macroscopic assessment**

| Category | Points | |
| --- | --- | --- |
| **Degree of defect repair**  In level with surrounding cartilage  75% repair of defect depth  50% repair of defect depth  25% repair of defect depth 0% repair of defect depth **Integration to border zone** Complete integration with surrounding cartilage  Demarcating border <1 mm  3/4^th^ of graft integrated, 1/4^th^ with a notable border >1 mm width  1/2 of graft integrated with surrounding cartilage, 1/2 with a notable border >1 mm From no contact to 1/4^th^ of graft integrated with surrounding cartilage  **Macroscopic appearance**  Intact smooth  Fibrillated surface Small, scattered fissures or cracs  Several, small or few but large fissure  Total degeneration of grafted area **Total maximum** | 4  3  2  1  0  4  3  2  1  0  4  3  2  1  0  12 |  |

**Table 3**

**Histological grading scale for cartilage repair**

| Category | Points | |
| --- | --- | --- |
| **Cell morphology**  Hyaline cartilage  Mostly hyaline cartilage  Mostly fibrocartilage  Mostly non-cartilage  Non-cartilage only  **Matrix-staining (metachromasia)** Normal (compared with host adjacent cartilage)  Slightly reduced  Markedly reduced  No metachromatic stain  **Surface regularity** Smooth (>3/4)  Moderate (>1/2-3/4)  Irregular (1/4-1/2)  Severely irregular (<1/4)  **Thickness of cartilage** >2/3  1/3-1/2  <1/3  **Integration of donor with host adjacent cartilage** Both edges integrated  One edge integrated  Neither edge integrated  **Total maximum** | 0  1  2  3  4  0  1  2  3  0  1  2  3  0  1  2  0  1  2  14 |  |
